# Alterations in One-Carbon Metabolism in Celiac Disease

**DOI:** 10.3390/nu12123723

**Published:** 2020-12-02

**Authors:** Rafael Martín-Masot, Natàlia Mota-Martorell, Mariona Jové, José Maldonado, Reinald Pamplona, Teresa Nestares

**Affiliations:** 1 Pediatric Gastroenterology and Nutrition Unit, Hospital Regional Universitario de Málaga, 29011 Malaga, Spain; rafammgr@gmail.com; 2 Department of Experimental Medicine, Lleida Biomedical Research Institute (IRBLleida), University of Lleida (UdL), 25198 Lleida, Spain; nataliamotamartorell@gmail.com (N.M.-M.); mariona.jove@udl.cat (M.J.); reinald.pamplona@udl.cat (R.P.); 3 Department of Pediatrics, University of Granada, 18071 Granada, Spain; jmaldon@ugr.es; 4 Pediatric Gastroenterology and Nutrition Unit, Hospital Universitario Virgen de las Nieves, 18071 Granada, Spain; 5 Instituto de Investigación Biosanitaria (ibs), 18071 Granada, Spain; 6 Maternal and Child Health Network, Carlos III Health Institute, 28029 Madrid, Spain; 7 Biomedical Research Centre (CIBM), Department of Physiology and “José MataixVerdú” Institute of Nutrition and Food Technology (INYTA), University of Granada, 18071 Granada, Spain

**Keywords:** amino acids, choline, celiac disease, folate cycle, mass spectrometry, methionine cycle, methionine savage pathway, transsulfuration pathway

## Abstract

Celiac disease (CD) is an autoimmune enteropathy associated with alterations of metabolism. Metabolomics studies, although limited, showed changes in choline, choline-derived lipids, and methionine concentrations, which could be ascribed to alterations in one-carbon metabolism. To date, no targeted metabolomics analysis investigating differences in the plasma choline/methionine metabolome of CD subjects are reported. This work is a targeted metabolomic study that analyzes 37 metabolites of the one-carbon metabolism in 17 children with CD, treated with a gluten-free diet and 17 healthy control siblings, in order to establish the potential defects in this metabolic network. Our results demonstrate the persistence of defects in the transsulfuration pathway of CD subjects, despite dietary treatment, while choline metabolism, methionine cycle, and folate cycle seem to be reversed and preserved to healthy levels. These findings describe for the first time, a metabolic defect in one-carbon metabolism which could have profound implications in the physiopathology and treatment of CD.

## 1. Introduction

Celiac disease (CD) is an autoimmune enteropathy, with a chronic and systemic trait, triggered by dietary gluten and related-prolamins in individuals that possess a genetic susceptibility [1,2]. Therefore, CD results from an interplay between genetic and environmental factors [3]. The affected subjects—usually children, but each time more diagnosed in adults—display different degrees of intestinal inflammation, ranging from a lymphocytic enteritis to crypt hyperplasia, severe infiltration of the lamina propria by mononuclear cell, and evident villous atrophy [4]. In agreement with these changes, the clinical manifestations range from asymptomatic to signs and symptoms of general malabsorption. Indeed, untreated symptomatic CD is associated with a relevant morbidity and mortality [5]. Thus, it can be associated with CD neurological diseases, osteoporosis, T-cell lymphoma, as well as other autoimmune diseases, such as type I diabetes, tiroiditis, and Addison’s disease [5]. Recent studies demonstrate that this initially considered rare disease is relatively common, affecting around 1–3% of the general population in both Europe and North America [6,7,8]. The genetic component of CD is associated with specific class II Major Histocompatibility Complex (MHC) alleles. Thus, more than 90% of CD subjects have the HLA-DQ2 allele, with DQ8 being the most common allele in the remaining patients; importantly, non-HLA genes were found to contribute to the disease [9]. However, and surprisingly, about 40–50% of the European general population is positive for the HLA–DQ2 heterodimer [10], clearly suggesting that other factors are needed for the development of the CD. Notably, there is a manifest of histological and clinical recovering when a strict gluten-free diet (GFD) is applied [1].

Metabolic changes seem to also be present in CD. Thus, metabolomics studies, despite being very limited and restricted to the use of nuclear magnetic resonance (NMR)-based methods, suggest minor but significant alterations in energy metabolism, lipid metabolism, and microbiome-derived metabolites [11,12,13,14,15,16,17]. Notably, different concentrations of methionine, choline, and choline-derived lipids in CD patients are described [11,12,13,15,16], suggesting a potential affectation of one-carbon metabolism. However, these observations were not discussed and there is a lack of additional details.

**Figure 1:**
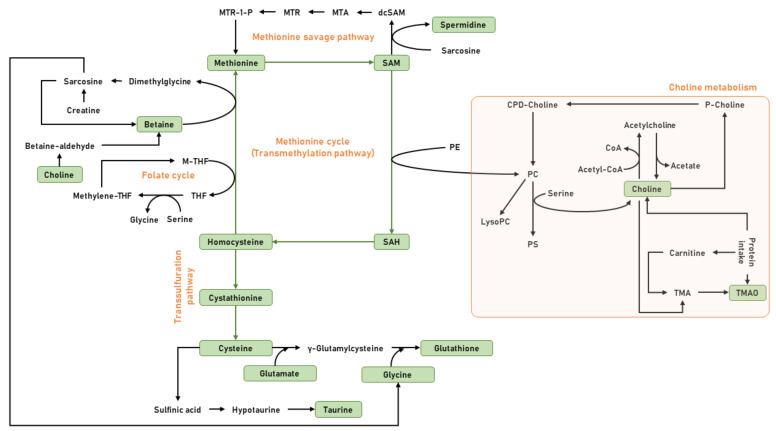
General view of the one-carbon metabolism. Choline, an essential micronutrient, can be obtained from exogenous sources—for instance, from foods such as eggs, beans, fish, nuts, seeds, and whole grain-, and from endogenous biosynthesis. The de novo synthesis of choline is catalyzed by the enzymatic activity of phosphatidylethanolamine-methyltransferase (PEMT) via the sequential methylation of PE, using S-adenosylmethionine (SAM) as a methyl donor. The oxidation of choline into its metabolite betaine by choline oxidase plays a role in the formation of methionine and SAM, methyl-donors that are involved in methylation pathways. SAM is considered the main methyl-donor for histone methyltransferases (HMTs) and DNA methyltransferases (DNMTs), key enzymes that catalyze both histone and DNA methylation. Choline can also be acetylated into acetylcholine (Ach) by the enzymatic activity of choline acetyltransferase (ChAT). Methionine is central in a complex metabolic pathway that can be divided into three parts—methionine cycle, the transsulfuration pathway, and polyamine biosynthesis. Thus, methionine is converted to the universal methyl donor SAM, which upon donation of one methyl group is converted to S-adenosylhomocysteine (SAH). SAH is hydrolyzed into homocysteine, which can be used to regenerate methionine via the betaine or folate cycle. In addition, homocysteine can be remethylated to methionine via folate cycle, or can also enter into the transsulfuration pathway, and be sequentially converted into cystathionine and cysteine, in a series of reactions catalyzed by enzymes that use vitamin B6 as cofactor. Finally, this cysteine can be directed to the glutathione synthesis onto the synthesis of taurine.

Choline and methionine, as well as other micronutrients such as folate, B vitamins, and betaine, participate in a complex metabolic network known as one-carbon metabolism [17,18,19]. Functionally, one-carbon metabolism is involved in the transfer of one-carbon moieties for the biosynthesis of phospholipids, amino acids, and DNA, as well as the methylation of proteins, RNA, and DNA. More specifically, this network comprises choline metabolism, folate cycle, methionine cycle or the transmethylation pathway, methionine savage pathway, and transsulfuration pathway (Figure 1).

As a consequence of these observations, it is plausible to postulate that CD patients have potential alterations in one-carbon metabolism. To date, no targeted metabolomics analysis investigating the differences in the plasma choline/methionine metabolome of CD subjects are reported. For this reason, we designed a study to detect and quantify a panel of metabolites, including 37different molecular species in 34 subjects, 17 CD patients, and 17 healthy control siblings. The CD patients are children treated with a GFD. This election is based on the assumption that GFD induces a reversion of CD symptoms and histological changes, as well as metabolic changes, and potential remaining or persistent metabolic alterations should be, if present, specific to the CD condition. Blood plasma was elected as source of information because it is easy to obtain, but also because it is the major carrier of metabolites in the body [20], and the composition of this biological fluid is well-known, even if it is in continuous change, reflecting the physiological states in health and disease [21,22], allowing the identification of new mechanistic pathways or targets that might lead to healthier states.

The metabolites selected and analyzed in the present study were—(a) methionine and its related metabolites, including the intermediates of the transmethylation pathway SAM, SAH, and homocysteine; betaine and spermidine as metabolites involved in the regeneration of methionine plasma levels; the intermediates of the transsulfuration pathway cysteine and cystathionine; taurine and glutathione as downstream metabolites of the transsulfuration pathway; and vitamin B6 metabolites pyridoxal, pyridoxal-5′-phosphate and pyridoxamine, as cofactors of the transsulfuration enzymes; (b) additional amino acids including 7 non-polar amino acids (alanine, glycine, leucine/isoleucine, phenylalanine, proline, tyrosine and valine), 4 polar uncharged amino acids (asparagine, serine, threonine and tryptophan), 1 polar negatively charged amino acid (glutamate), and 2 polar positively charged amino acids (arginine and histidine); (c) TCA cycle metabolites, including pyruvate, citrate, isocitrate, α-ketoglutarate, succinate, fumarate, and malate; and (d) methionine-derived lipid intermediates such as choline and TMAO. The plasma metabolites profile was determined using an LC–MS/MS platform to systematically define the specific phenotypic patterns associated with the genotypes of CD.

## 2. Materials and Methods 

### 2.1. Subjects

This work was a cross-sectional study in children with CD (*n* = 17) and healthy control siblings (*n* = 17) between 4 and 17 years of age. Children with CD were divided according to time, on a GFD. The recruitment was carried out from January to December of 2018, attending the Gastroenterology, Hepatology, and Child Nutrition Service from the “Virgen de las Nieves” University Hospital in Granada, Spain. Infants with CD were diagnosed according to the European Society for Pediatric Gastroenterology Hepatology and Nutrition (ESPGHAN) criteria [23]. Infants with liver or kidney diseases, inflammatory bowel disease, diabetes, chronic asthma, and consumption of dietary supplements containing substances with antioxidant activity were excluded. We also excluded obese patients (according to the criteria of the International Task Force) [24]. Written informed consent was obtained from all parents and those who did not sign it were excluded. The study was approved by the Ethics Committee of the University of Granada (Ref. 201202400000697) and complied with the Good Clinical Practice guidelines.

### 2.2. Clinical and Socio-Demographics

Participants’ clinical and socio-demographic characteristics, including adherence to GFD, were assessed by the same group of researchers.

### 2.3. Anthropometric Measures

Anthropometric characteristics (weight, height) were assessed in the siblings and the celiac subjects. Height was measured to the nearest 5 mm, using a stadiometer (Seca 22, Hamburg, Germany). Body weight was measured using the same mechanical balance (Seca200, Hamburg, Germany).

### 2.4. Blood Samples

Blood samples were obtained by venipuncture in the morning (between 7 and 8 AM) after fasting overnight (8–10 h), and collected in one vacutainer CPT (Cell Preparation Tube; BD, Franklin Lakes, NJ, USA) containing sodium heparin as the anticoagulant. Plasma fractions were collected after blood sample centrifugation, and immediately frozen in liquid nitrogen, and transferred before 4 h to a −80 °C freezer for storage, to be used later for metabolomic analyses.

### 2.5. Targeted Metabolomics

#### 2.5.1. Chemicals 

Unless otherwise Specified, All Reagents were from Sigma-Aldrich, and of the Highest Purity Available.

#### 2.5.2. Sample Processing

Plasma metabolites extraction was performed based on the methodology previously described (Method 1 [25]). In brief, 10 µL of plasma were added to 30 µL of cold methanol containing 1 μg/mL of Phe-13C as the internal standard and 1 μM BHT as antioxidant. Then, the samples were incubated at room temperature for 15 min and centrifuged at 12,000× *g* for 3 min. Finally, the supernatant was filtrated through a 0.22-μm organic diameter filter (CLS8169, Sigma, Madrid, Spain) and transferred to Agilent (Barcelona, Spain) vials, with glass inserts, for further analysis.

Sulphur-containing metabolites were extracted on the basis of the methodology previously described (Method 2 [26]). In brief, 2 µL of 5% DTT diluted in methanol (m/v) were added to 10 µL of plasma. The resulting solution was vortexed for 1 min and allowed to stand at room temperature for 10 min. For protein precipitation, 40 µL of acetonitrile containing 0.1% formic acid (*v*/*v*), 0.05 % trifluoroacetic acid (*v*/*v*), and 1 µg/mL of Phe-13C as the internal standard was added to the sample, and the solution was vortexed for 2 min. Then, the samples were incubated at room temperature for 15 min and centrifuged at 12,000× *g* for 3 min. Finally, the supernatant was filtrated through a 0.22-μm organic diameter filter (CLS8169, Sigma, Madrid, Spain) and transferred to Agilent (Barcelona, Spain) vials, with glass inserts, for further analysis.

#### 2.5.3. Analysis Conditions

For the non-sulfur-containing metabolites, 2 µL of the extracted sample was injected, based on the method described (Method 1 [25]). Chromatographic separation was achieved on a reversed-phase column (Zorbax SB-Aq 2.1 × 50 mm, 1.8 µm particle size, Agilent Technologies, Barcelona, Spain) equipped with a pre-column (Zorbax SB-C8 Rapid Resolution Cartridge, 2.1 × 30 mm, 3.5 µm particle size, Agilent Technologies, Barcelona, Spain), with a column temperature of 60 °C. The flow rate was 0.6 mL/min during 19 min. Solvent A was composed of water containing 0.2% acetic acid (*v*/*v*) and solvent B was composed of methanol containing 0.2% acetic acid (*v*/*v*). The gradient started at 0% of solvent B and increased to 10% B in 5 min, 10% of solvent B increased to 100% B in 3 min, and held for 1 min. Post-time was established in 5 min. Electrospray ionization was performed in both positive and negative ion mode (depending on the target metabolite), using N2 at a pressure of 50 psi for the nebulizer, with a flow of 12 L/min and a temperature of 325 °C, respectively.

For the sulfur-containing metabolites, 10 µL of the extracted sample was injected, based on the method described (Method 2 [26]). Chromatographic separation was achieved on a reversed-phase Supelcosil LC-CN column (Supelco of 4.6 × 250 mm, 5 µm particle size, Sigma, Madrid, Spain), with a column temperature of 30 °C. The flow rate was maintained at 0.5 mL/min during 10 min, using a mobile phase of 10:90 acetonitrile/water with 0.1% formic acid (*v*/*v*). Electrospray ionization was performed in both positive and negative ion mode (depending on the target metabolite) using N2 at a pressure of 50 psi for the nebulizer, with a flow of 12 L/min and a temperature of 325 °C, respectively.

The individual conditions for the detection and quantification of plasma metabolites are listed in Table 1. The analysis was performed through liquid chromatography coupled to a hybrid mass spectrometer, with electrospray ionization and a triple quadrupole mass analyzer. The liquid chromatography system was an ultra-performance liquid chromatography model 1290 coupled to an LC-ESI-QqQ-MS/MS model 6420, both from Agilent Technologies (Barcelona, Spain). Data were collected using the MassHunter Data Analysis Software (Agilent Technologies, CA, USA). Samples were decoded and randomized before injection. Metabolite extraction quality controls (plasma samples with internal Phe-13C) were injected every 10 samples. Peak determination and peak area integration were carried out with MassHunter Quantitative Analyses (Agilent Technologies, CA, USA). Peak area was normalized by internal Quantitative Analyses (Agilent Technologies, CA, USA). Peak area was normalized by internal Phe-^13^C.

### 2.6. Data Analyses

We used the mean and standard deviation for the quantitative variables and percentage of participants (%) for categorical variables, to describe the baseline characteristics of the population. We conducted Student’s *t*-test or Welch-test to explore the differences in the continuous variables. Furthermore, we assessed differences in categorical variables by using the chi-squared test.

Multivariate statistics were performed using the Metaboanalyst software on transformed data (auto-scaled and log-transformed). Univariate statistics were performed using R version 3.6.1. Paired-*t*-test was used to measure sibling differences. Minimum signification level was set at *p* < 0.05. Plots were performed using the GraphPad Prism (v8.0.1).

## 3. Results

### 3.1. Clinical Characteristics

The baseline clinical characteristics of the study sample are shown in Table 2. A total of 34 children participated in the study; 17 CD and 17 healthy siblings. There were no differences between groups, including factors that could affect metabolomic analysis, like lifestyle (physical activity and dietary habits).

### 3.2. Targeted Metabolomic

A targeted metabolomic approach to analyze 7 energetic metabolism intermediates (Table 3), 12 amino acids (Table 4), and 18 metabolites related to methionine and choline metabolism (Table 5), in CD patients (17) and healthy siblings (17) was performed. 

**Figure 2:**
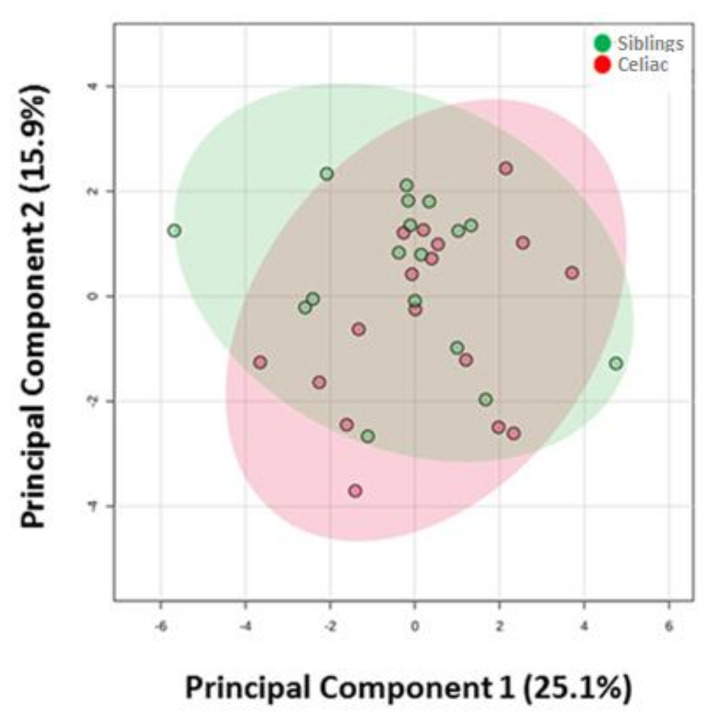
Principal Component Analyses shows no differences in plasma metabolome of metabolites analyzed between celiac disease patients and healthy siblings.

First, and in order to have a global view of how CD affects these 37 metabolites, a multivariate statistics approach was applied. As Figure 2 shows, Principal Component Analyses (PCA) did not reveal the existence of a plasma-specific metabolic profile associated with methionine and choline metabolism, amino acids, or energetic metabolism intermediates for celiac individuals (Figure 2). Furthermore, no similar metabolomics profile between siblings was observed.

Second, and in order to specifically determine whether the plasma levels of each metabolite were affected by the CD condition, a univariate statistic (Paired T-Test) was applied. In line with the multivariate statistics, this analysis confirmed small changes in the metabolome analyzed. Specifically, no differences were found for plasma energetic metabolism intermediates (Table 3), and the plasma amino acids (Table 4) between CD children and healthy siblings.

**Figure 3:**
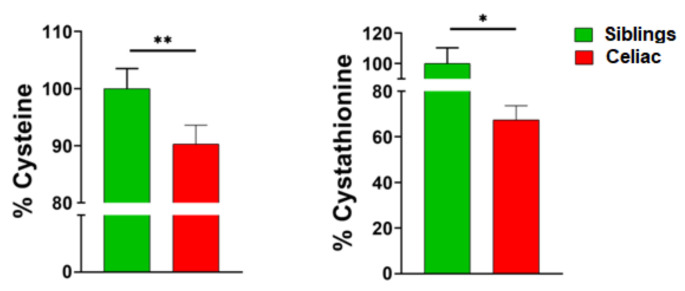
Plasma cysteine and cystathionine content is decreased in celiac disease children as compared to healthy siblings. * *p* < 0.05, ** *p* < 0.01.

When we focused on choline and methionine metabolism, we observed similar results. Thus, any difference was verified for metabolites involved in choline metabolism, folate cycle, methionine savage pathway, and methionine cycle (Table 5). In contrast, differences were observed in the transsulfuration pathway. More specifically, univariate statistics revealed a reduction of cysteine and cystathionine plasma content (10% and 30%, respectively) in CD individuals, as compared to healthy siblings (Figure 3).

## 4. Discussion

The genetic, immunological, and environmental components of the CD condition determines the variability in the clinical expression of this pathology. The variety of symptoms and signs induced by the recent findings of CD suggest that metabolic alterations in subjects with CD must be added. Although today in most cases the diagnosis of CD requires confirmation that the disease has a positive biopsy, a better knowledge of the metabolic processes underlying this pathology could offer the opportunity to uncover potential new biomarkers, which could be useful for both the diagnosis of doubtful cases due to the histological results, as well as for the follow-up of the disease. 

At present, a very limited number of metabolomics studies of CD are available, but they seem to verify the existence of minor but significant metabolic differences between healthy individuals and celiac patients (children and adults) [11,12,13,14,15,16]. In these studies, using NMR or mass spectrometry-based methods, different biological sample types including serum, plasma, urine, and fecal material were analyzed. On the whole, the metabolomics fingerprint found derives from alterations in gut microflora or intestinal permeability, malabsorption, and alterations in metabolism [11,12,13,14,15,16]. Notably, the introduction of a strict GFD seems to reverse the metabolic profile of CD patients to the healthy condition. The main observed differences in serum metabolites between CD patients and siblings were lower levels of amino acids, such as asparagine, isoleucine, proline, valine and methionine, and diverse metabolites such as methylamine, pyruvate, creatinine, choline, methylglutarate, lactate, and lipids (phosphatidycholines), and higher levels of glucose, 3-hydroxybutyric acid, and triacylglycerides [11,12,13,14,15,16]. The decreased levels of choline, methionine, and lipids (particularly phosphatidylcholines) could be relevant because these metabolites could express defects in the one-carbon metabolism. Our findings confirm this idea, uncovering the transsulfuration pathway as potentially affected by CD, while the other pathways involved in one-carbon metabolism seems to be preserved.

Considering the transsulfuration pathway, the decreased concentration of cysteine and cystathionine in CD patients could be due to defects in the enzymes that participate in its biosynthesis. Thus, the step from homocysteine to cystathionine is mediated by cystathioninebeta-synthase (CBS; EC 4.2.1.22), and the conversion of cystathionine to cysteine is catalyzed by the enzyme cystathionine gamma-lyase or cystathionase (CTH; EC 4.4.1.1) [27,28,29]. Interestingly, both enzymes are cytoplasmic homotetramers that require vitamin B6 (pyridoxal phosphate) as a cofactor, and both are able to produce hydrogen sulfide (H2S), a gasotransmitter, by using cysteine [28,29,30,31]. The fact that no differences were observed for glutathione and vitamin B6 content in CD children, suggests a specific defect at the level of these enzymes. Consequently, in addition to potential problems derived from deficiencies in cysteine content, defects in hydrogen sulfide synthesis could be especially relevant in CD patients, due to the important physiological processes regulated by this gaseous molecule. Thus, H2S modulates several aspects of cellular physiology such as proteostasis, antioxidant defense, oxygen sensing, mitochondrial function, inflammation, and second messenger signaling, as well as of system physiology affecting cardiovascular, nervous, and immune functions [32,33,34]. Remarkably, H2S seems to play a key regulatory role in mitochondrial energy production [35]. Therefore, alterations in H2S synthesis in CD subjects could be associated with defects in energy metabolism, as it was in fact demonstrated in metabolomics studies showing lower content in energy metabolites [11,12]. More studies, however, are needed to confirm and develop this idea in CD.

A possible alternative or additional interpretation is that a mutation in the SAH hydrolase (compatible with higher levels of SAH, not significant in this study) could lead to reduced levels of HCys (not significant in this study), and therefore, reduced cystathione and Cys. According to Malacards [36], SAH hydrolase deficiency causes symptoms like failure to thrive, abnormal teeth, elevated transaminases (that are seen in CD patients), high creatine kinase [37], or hypermethioninemia (that is common in newly diagnosed CD and tends to normalize under GFD).

The present work has some limitations—(1) the present study sample size was relatively small, and, consequently, the present results must be interpreted with caution. The fact that 5 of the celiac children were on a GFD for less than 12 months could influence the results, since a long-term GFD could act on the metabolic pathways after 12 months, as other studies showed in an adult population [11]. On the other hand, there are no large metabolomic study population and this is the first siblings metabolomic approach in pediatric CD. Moreover, the fact that the study participants are matched with their siblings, reduces the differences that can occur in metabolomic processes [38], which could be influenced by various mechanisms, like perform a GFD, so this fact would decrease the variability, since the majority of families with a member with CD broadly adhere to this type of diet [39]. (2) Plasma samples were obtained after overnight fasting, in order to avoid the effect of the diet in plasma metabolome. However, we cannot discard that the differences seen in the transsulfuration pathway could reflect other alterations not directly related to this pathway. (3) Not all one-carbon metabolism metabolites could be detected and analyzed, so it must be borne in mind that the view we obtained of this metabolic pathway was not complete. (4) Cysteine and Cystationine FDR adjusted *p*-value was higher than 0.05, so further studies are needed to confirm our results. (5) Although the difference was not statistically significant, there were differences in both sex and age and it is known that many metabolites could vary according it.

In conclusion, the data presented here, suggest for the first time, significant alterations in the transsulfuration pathway of CD subjects despite being under a GFD, as a result of expression of a new physiopathological mechanism underlying the CD pathology, which needs further studies to be verified.

## Figures and Tables

**Table 1:** Analytical traits of the metabolites measured in plasma.

Compound	Precursor Ion	Product Ion	Fragmentor	CE	CAV	RT	RT Window	Polarity	Extraction	Acquisition Method
**Amino acids**										
Alanine	90.06	44.2	40	8	7	0.376	2	Positive	Methanol	1
Arginine	175.1	70.2	60	20	7	0.320	2	Positive	Methanol	1
Arginine	175.1	60.2	60	15	7	0.320	2	Positive	Methanol	1
Asparagine	133	74.1	60	15	7	0.376	2	Positive	Methanol	1
Aspartate	134	43.2	60	15	7	0.362	2	Positive	Methanol	1
Aspartate	132	88.1	60	15	7	0.362	2	Negative	Methanol	1
Glutamate	146	102.1	60	15	7	0.363	2	Negative	Methanol	1
Glutamate	146	41	60	15	7	0.363	2	Negative	Methanol	1
Glycine (ND)	76.04	48	40	0	7	0.340	2	Positive	Methanol	1
Glycine (ND)	76.04	30	40	4	7	0.340	2	Positive	Methanol	1
Histidine	156	110.1	60	15	7	0.320	2	Positive	Methanol	1
Histidine	156	56.2	60	25	7	0.320	2	Positive	Methanol	1
Leucine/Isoleucine	132.1	86	64	8	7	0.591	2	Positive	Methanol	1
Leucine/Isoleucine	132.1	69	64	16	7	0.591	2	Positive	Methanol	1
Phenylalanine	164	147	100	15	7	0.841	2	Negative	Methanol	1
Phenylalanine	164	103.1	100	15	7	0.841	2	Negative	Methanol	1
Proline	116	70.2	60	15	7	0.392	2	Positive	Methanol	1
Serine	106.05	60	64	8	7	0.350	2	Positive	Methanol	1
Serine	106.05	42	64	24	7	0.350	2	Positive	Methanol	1
Serine	104.03	74	64	8	7	0.350	2	Negative	Methanol	1
Threonine	120	74.2	60	15	7	0.358	2	Positive	Methanol	1
Threonine	120	56.2	60	15	7	0.358	2	Positive	Methanol	1
Tryptophan	205	188.1	60	15	7	1.230	2	Positive	Methanol	1
Tryptophan	205	146.1	60	15	7	1.230	2	Positive	Methanol	1
Tyrosine	180.1	163.1	100	15	7	0.548	2	Negative	Methanol	1
Tyrosine	180.1	119.1	100	15	7	0.548	2	Negative	Methanol	1
Valine	118.08	72	64	8	7	0.430	2	Positive	Methanol	1
Valine	118.08	55	64	20	7	0.430	2	Positive	Methanol	1
**Methionine metabolism**										
Betaine	118.09	59.2	136	16	7	0.425	2	Positive	Methanol	1
Betaine	118.09	58.2	136	32	7	0.425	2	Positive	Methanol	1
Cysteine	122.02	76	64	12	7	6.312	2	Positive	ACN-DTT	2
Cysteine	122.02	59	64	24	7	6.312	2	Positive	ACN-DTT	2
Cystathionine	223.07	134	88	8	7	6.818	2	Positive	ACN-DTT	2
Cystathionine	223.07	88	88	28	7	6.818	2	Positive	ACN-DTT	2
Glutathione	308.09	179	88	8	7	0.500	2	Positive	ACN-DTT	1
Glutathione	308.09	76	88	24	7	0.500	2	Positive	ACN-DTT	1
Homocysteine	136.18	90.1	135	15	7	7.225	2	Positive	ACN-DTT	2
Homocysteine	136.18	56.2	135	15	7	7.225	2	Positive	ACN-DTT	2
Methionine	150.05	104	64	4	7	0.480	2	Positive	ACN-DTT	1
Pyridoxal	168.05	150	64	8	7	0.522	2	Positive	Methanol	1
Pyridoxal	168.05	94	64	24	7	0.522	2	Positive	Methanol	1
PLP (Pyridoxal-5′-P)	248.03	150	112	12	7	0.700	2	Positive	Methanol	1
PLP (Pyridoxal-5′-P)	248.03	67	112	32	7	0.700	2	Positive	Methanol	1
Pyridoxamine	169.09	152	64	8	7	0.366	2	Positive	Methanol	1
Pyridoxamine	169.09	134	64	20	7	0.366	2	Positive	Methanol	1
SAH	385.1	136	112	20	7	1.130	2	Positive	Methanol	1
SAH	385.1	88	112	48	7	1.130	2	Positive	Methanol	1
SAM	399.1	250	112	12	7	0.396	2	Positive	Methanol	1
SAM	399.1	136	112	28	7	0.396	2	Positive	Methanol	1
Spermidine	146.1	84	88	24	7	0.300	2	Positive	Methanol	1
Spermidine	146.1	72	88	12	7	0.300	2	Positive	Methanol	1
Taurine	126.02	108	88	8	7	0.380	2	Positive	Methanol	1
Taurine	124	80	112	20	7	0.380	2	Negative	Methanol	1
**TCA cycle intermediates**										
α-Ketoglutarate	145.01	101	64	4	7	0.435	2	Negative	Methanol	1
α-Ketoglutarate	145.01	57	64	20	7	0.435	2	Negative	Methanol	1
Citrate	191.01	111	88	8	7	0.637	2	Negative	Methanol	1
Citrate	191.01	87	88	16	7	0.637	2	Negative	Methanol	1
Fumarate	115.01	71	64	4	7	0.550	2	Negative	Methanol	1
Fumarate	115.01	27	64	4	7	0.550	2	Negative	Methanol	1
Isocitrate	191.01	111	88	8	7	0.393	2	Negative	Methanol	1
Isocitrate	191.01	87	88	16	7	0.393	2	Negative	Methanol	1
Malate	133.02	115	64	8	7	0.400	2	Negative	Methanol	1
Malate	133.02	71	64	12	7	0.400	2	Negative	Methanol	1
Pyruvate	87.01	43	64	4	7	0.413	2	Negative	Methanol	1
Succinate	117.02	73	64	8	7	0.570	2	Negative	Methanol	1
Succinate	117.02	55	64	20	7	0.570	2	Negative	Methanol	1
**Lipid intermediates**										
Choline	104.11	60.2	112	16	7	0.390	2	Positive	Methanol	1
TMAO	76.08	59.2	64	8	7	0.400	2	Positive	Methanol	1
TMAO	76.08	58.2	64	20	7	0.400	2	Positive	Methanol	1
**ISTD**										
PheC13	167.09	120.1	70	8	7	0.870	2	Positive	Methanol/ACN-DTT	1/2
PheC13	167.09	77	70	44	7	0.870	2	Positive	Methanol/ACN-DTT	1/2
PheC13	167.09	103	70	28	7	0.870	2	Positive	Methanol/ACN-DTT	1/2
PheC13	167.09	51.1	70	60	7	0.870	2	Positive	Methanol/ACN-DTT	1/2

Fragmentor, collision energy (CE), and cell acceleration voltage (CAV) are given as voltage; retention time (RT) and RT window in minutes; and product and precursor ion as m/z. For the acquisition method details see the methods section, and references for Method 1 [25] and 2 [26]. ND = Not detected metabolite, concentration below LOD.

**Table 2:** Sociodemographic characteristics of the study participants.

Variable	Healthy Siblings (*n* = 17)	Celiac Children (*n* = 17)	*p-*Value
Age (years)	11.25 (4.23)	9.39 (2.77)	0.145
Sex (female, n [%])	10 (58.8)	13 (76.4)	0.271
Weight (kg)	38.54 (16.9)	30 (9.63)	0.082
Height (cm)	140.66 (20.32)	131.2 (19.56)	0.178
BMI (kg/m2)	18.5 (3.98)	17 (1.58)	0.166
Moderate physical activity (min/week)	69.64 (37)	81.4 (56.9)	0.52
Mediterranean Diet adherence n (%)			
Low	1 (5.9)	1 (5.9)	
Medium	8 (47.1)	7 (41.2)	0.936
High	7 (41.2)	8 (47.1)	
Diet			
Less than 12 months on a GFD		5	
More than 12 months on a GFD		12	
HLA DR-DQ genotype			
Negative	5	0	
HLA-DQ2+	11	14	
HLA DQ8+	0	0	
HLA-DQ2+DQ8+	1	3	

SD: standard deviation; Values shown as mean (standard deviation) unless otherwise indicated; MD: Mediterranean Diet; and GFD: gluten-free diet.

**Table 3:** Plasma energetic metabolism intermediates content in celiac patients and healthy siblings.

Metabolite	Healthy Siblings	Celiac Children	Paired T-Test
α-Ketoglutarate	100 ± 4.2	95.7 ± 3.8	0.282
Citrate	100 ± 6.7	101.4 ± 6.3	0.713
Fumarate	100 ± 4.6	101.5 ± 3.7	0.684
Isocitrate	100 ± 7.5	104.4 ± 7.1	0.471
Malate	100 ± 6.4	101.7 ± 5.7	0.776
Pyruvate	100 ± 6.9	97.2 ± 10	0.720
Succinate	100 ± 5.4	99.4 ± 4.3	0.909

Values were expressed as % with respect to siblings.

**Table 4:** Plasma amino acids content in celiac patients and healthy siblings.

Amino Acid	Healthy Siblings	Celiac Children	Paired T-Test
Ala	100 ± 4.2	96.6 ± 7.4	0.621
Arg	100 ± 6.1	92.9 ± 5.5	0.385
Asn	100 ± 3.3	103.6 ± 3.9	0.376
Asp	100 ± 4.8	109.9 ± 5.8	0.085
His	100 ± 3	100.2 ± 2	0.938
Leu/Ile	100 ± 5	96.1 ± 4.5	0.515
Phe	100 ± 3.9	96.6 ± 4.1	0.334
Pro	100 ± 8.5	90.2 ± 6.4	0.283
Thr	100 ± 6.5	100.1 ± 6	0.995
Try	100 ± 4	93.4 ± 3.5	0.231
Tyr	100 ± 8.8	100.3 ± 7.1	0.967
Val	100 ± 5.2	99.7 ± 3.9	0.965

Values were expressed as % with respect to siblings.

**Table 5:** Plasma metabolite variations in healthy siblings and celiac children.

Metabolite	Healthy Siblings	Celiac Children	Paired T-Test
Betaine	100 ± 6.4	93.7 ± 3.8	0.249
Choline	100 ± 5.2	95.1 ± 6.4	0.468
Cys	100 ± 3.5	90.3 ± 3.4	0.008
Cystathionine	100 ± 10.4	67.4 ± 6.2	0.024
GSH	100 ± 7.2	110.9 ± 10.1	0.368
Glu	100 ± 14.3	109.7 ± 13.3	0.611
Gly	100 ± 43.3	101.3 ± 27.8	0.982
Homocysteine	100 ± 12.7	86.4 ± 9.1	0.266
Met	100 ± 4.6	94.9 ± 5.6	0.360
PLP	100 ± 18.4	105.7 ± 18.1	0.485
Pyridoxal	100 ± 10.8	108.2 ± 11.8	0.123
Pyridoxamine	100 ± 5.6	94.4 ± 6.3	0.244
SAH	100 ± 6.5	134.5 ± 22.2	0.158
SAM	100 ± 20.1	89.1 ± 19.9	0.282
Ser	100 ± 4.4	103 ± 3.5	0.519
Spermidine	100 ± 28.6	105.3 ± 28.4	0.186
Taurine	100 ± 16.2	105.9 ± 17	0.576
TMAO	100 ± 30.6	66.1 ± 21.7	0.329

Values were expressed as % with respect to control individuals.
